# Health-related quality of life by allergy symptoms in elementary school students

**DOI:** 10.1186/s12955-018-0922-y

**Published:** 2018-05-15

**Authors:** Sang-Kyu Kim, Min-Woo Jo, Seon-Ha Kim

**Affiliations:** 1 Department of Preventive Medicine, Dongguk University College of Medicine, 123, Dongdae-ro, Gyeongju-si, Gyeongsangbuk-do 38066 Republic of Korea; 2 Department of Preventive Medicine, University of Ulsan College of Medicine, 88, Olympic-ro 43-gil, Songpa-gu, Seoul, 05505 Republic of Korea; 3 Department of Nursing, Dankook University College of Nursing, 119, Dandaero, Dongam-gu, Cheonan, Chungnam 31116 Republic of Korea

**Keywords:** Allergic rhinitis, Atopic dermatitis, Asthma, Child, EQ-5D-Y, Health related quality of life

## Abstract

**Background:**

Globally, allergic diseases are very common in childhood and may affect children’s quality of life. This study aimed to explore health-related quality of life of elementary school students with allergy symptoms using the EQ-5D-Y and to examine the validity and feasibility of the EQ-5D-Y.

**Methods:**

The study subjects were the students within 50 elementary schools which voluntarily participated in research project. In this sample population, the EQ-5D-Y questionnaire was self-administered by the students, and demographic and health information were collected from the student’s parents. The parents’ information was used to investigate the proportion of students with allergic symptoms (wheezing, runny or blocked nose, and itchy rash) in the past 12 months. In addition, we analyzed the correlation of symptom reporting and EQ-5D-Y including EQ-VAS.

**Results:**

The overall return was 9117 responses, of which 198 (2.2%) lacked responses on the EQ-5D-Y dimension and 1258 (13.8%) on the VAS score. There were significant differences in symptom reporting in all EQ-5D-Y dimensions between groups with or without allergic symptoms. Particularly, there was a large difference in reporting rates in ‘having pain or discomfort’ and ‘feeling worried, sad or unhappy’ dimensions. As the number of allergic symptoms increased, in all dimensions also the problem reporting rate tended to increase.

**Conclusions:**

As expected, the presence of allergic symptoms is inversely correlated with the quality of life of children. The EQ-5D-Y instrument proved to be useful in terms of feasibility and construct validity in assessing the quality of life of Korean elementary school students.

## Background

Asthma, rhinitis, and atopic dermatitis in childhood are common chronic diseases worldwide including Korea [1, 2]. Globally, in 2013, in the 6-7 year old group, the prevalence of asthma was 11.7%, of rhinoconjunctivitis 8.5% and of atopic dermatitis 7.9%, although there were wide variations in the prevalence and severity according to the world region [1]. These allergic diseases not only increase the economic burden but also affect the quality of life of children, and their parents, respectively [2–6].

Interest in self-reported health-related quality of life [HRQOL] as an outcome measure in children and adolescents has grown in recent decades in both hospital and outpatient settings [7–9]. Consequently, a number of instruments have been developed to assess HRQOL in children and adolescents. Generic instruments can be applied to various population groups, allowing comparison of the relative effects of healthcare interventions and the quality of life between healthy and sick children [10]. Assessment tools for HRQOL for adults may not be appropriate for children, especially if the children’s language and cognitive abilities are not yet well enough developed. In addition, there may be some discrepancies between children’s self-report and proxy reports e.g. by parents [11–14].

The EuroQoL group developed a child-friendly version of the already established EQ-5D 3 level version [EQ-5D-3 L] [15], the EQ-5D for youth [EQ-5D-Y] [16], to assess HRQOL in children and adolescents [17]. The new instrument is simple and easy to self-administer. Previous studies supported the feasibility of EQ-5D-Y as an instrument with low missing or ambiguous answers [6, 18–24].

In Jelsma’s study comparing EQ-5D-Y and EQ-5D-3 L in children, the EQ-5D-Y led to fewer missing responses than the EQ-5D-3 L and response ranges in the EQ-5D-Y were broader than that in the EQ-5D-3 L, suggesting that the responsiveness in EQ-5D-Y may be better [19]. The validity [6, 18, 20–23, 25, 26], reliability [20–22, 26] and responsiveness [26] of the EQ-5D-Y were demonstrated in populations including children and adolescents with chronic diseases including asthma. The Korean version of the EQ-5D-Y was launched in 2015 by the EuroQol group, and HRQOL data for elementary school students obtained using the EQ-5D-Y instrument in Korea was published firstly in 2017 [24].

The purpose of this study was to explore health-related quality of life of elementary school students with allergy symptoms using the EQ-5D-Y and to examine the validity and feasibility of the EQ-5D-Y.

## Methods

### Study design and procedure

This study is a cross-sectional survey. The subjects of the study were students in 50 primary schools which voluntarily participated in research project in Gyeongsangbuk Province in eastern South Korea. All schools were general schools, that is, no special schools were included. Of the participating schools, 39 were located in the city and 11 were located in the countryside. The study started with 24 schools in 2015, and another 26 were added in 2016. Whereas in the former 24 schools only the first grade students participated in the survey, in the latter 26 schools the survey was conducted in all grades.

Written questionnaires for both children and parents, together with an informative letter and a consent form for the children as well as for their parents or guardians, were provided in sealed envelopes. These envelopes then were distributed in the classes by teachers, to be taken home by the children and to be filled at home. One copy of the written consent form was signed by both the parents/guardians, and the children, respectively.

The questionnaires consisted of three parts: 1) questions on socio-demographic data (i.e., gender, age, and grade), 2) the Korean-translated modified version of the International Study of Asthma and Allergies in Childhood (ISAAC) questionnaire [i.e., physician diagnosis of asthma, allergic rhinitis and atopic dermatitis since birth, treatment and symptoms of the asthma, allergic rhinitis and atopic dermatitis in the past 1 year, school absence due to asthma, allergic rhinitis and atopic dermatitis past 12 months, and emergency room (ER) visits due to asthma] [27], and 3) the EQ-5D-Y. Socio-demographic and health information was requested to be filled in by the parents/guardians, and the EQ-5D-Y was requested to be self-completed by the students. After the EQ-5D-Y questionnaire, information was requested regarding who actually completed the EQ-5D-Y, whether the parents, the students with the help of their parents, or the students themselves. The completed questionnaires were submitted to the school by students. It took from 2 to 7 days. No further reminders to reply were sent. The survey was conducted between February and September, 2016.

The study was approved by the Institutional Review Board of Dongguk University (110757-201,701-HR-02-03).

### EQ-5D-Y

The EQ-5D-Y consists of the EQ-5D-Y descriptive system and the EQ visual analogue scale (EQ-VAS). The EQ-5D-Y descriptive system asks a child to check its best description in each dimension about his or her health on the present day. The descriptive system comprises the same five dimensions as does the EQ-5D-3 L, except that it uses child-friendly wording (mobility, looking after myself, doing usual activities, having pain or discomfort, and feeling worried, sad or unhappy) to the target population 8 to 15 years old [16]. Each dimension has three response levels: ‘no,’ ‘some,’ and ‘a lot of’ problems in four of the dimensions, and ‘not,’ ‘a bit,’ and ‘very’ in the fifth dimension [25]. The additional EQ-VAS records the respondent’s self-rated health (“How good is your health TODAY?”) on a vertically numbered, visual analogue scale from 0 to 100. Its endpoints are labelled ‘The worst health you can imagine’ at 0 and ‘The best health you can imagine’ at 100 [25]. A health state summary descriptor is defined by recording the problem level from each of the five dimensions using the EQ-5D-Y. Each state is referred to by a 5-digit code. For example, the 11111 state represents a full health state with no problems in any dimension, whereas 11312 represents no problems with mobility or looking after oneself, a lot of problems with usual activities, no pain or discomfort, and being a bit worried, sad or unhappy [21]. Thus, with this questionnaire, 243 (3^5^) possible health states can be defined. The use of the EQ-5D-Y questionnaire in this study was approved by the EuroQoL group.

### Analysis

To evaluate the feasibility of the EQ-5D-Y, all returned questionnaires (*n* = 9117) were analyzed. However, 198 missed responses, which is not completing at least one dimension of the EQ-5D-Y, of the total replies were excluded to the primary analysis, including evaluation of the validity of the EQ-5D-Y. The feasibility of using the EQ-5D-Y to measure the HRQOL of Korean elementary school students was analyzed using the completion rate of the EQ-5D-Y questionnaires and the actual responders. We explored demographic characteristics and missing responses categorized by the person filling out the questionnaire (i.e., parents, the students with the help of their parents, and the students themselves).

To explore the construct validity of the EQ-5D-Y, the differences in reported problem rate in EQ-5D-Y dimensions and EQ-VAS score according to allergic symptoms (i.e., wheezing or whistling in the chest, runny or blocked nose, and itchy rash) were analyzed. Differences in problem reporting rates were tested with chi-square test and EQ-VAS score differences with independent t-test. In addition, the difference in the proportion of respondents reporting problem in EQ-5D-Y dimensions and their EQ-VAS score depending on the number of allergic symptoms reported were determined using chi-square test or ANOVA with post hoc Tukey test as appropriate. According to previous studies, the reporting rate in the group with allergic symptoms was expected to be higher than that of the group with no allergic symptoms, and the higher, the higher the problem reporting rate in EQ-5D-Y [5, 18, 20, 28, 29]. *P*-value of less than 0.05 was considered statistically significant. All statistical analyses were performed using SAS software version 9.1 (SAS Institute, Cary, NC, USA).

## Results

Of 9949 handed out questionnaires, 9117 (91.6%) came back to the researchers. The median school response rate was 95%, with a minimum of 68.8% and a maximum of 100%.

Demographic characteristics and health information of the students are in Table 1. The age of participants ranged from 7 to 13 years, with an average age of 10.2 years (SD, 1.8). Age differed significantly (*P* < 0.001) between valid responders (mean age = 10.2) and responders who did not complete EQ-5D-Y descriptive system (mean age = 9.0) (data not shown). The biggest proportion of respondents – some 25% – was in 1st grade, whereas each some 15% were in grades 2 through 6. Of the missing respondents, 125 (63.1%) were in the 1st grade. Over the past 12 months, wheezing or whistling in the chest (asthma) were reported by 259 (2.9%), runny or blocked nose (allergic rhinitis) by 2477 (27.8%), and itchy rash symptoms (atopic dermatitis) by 1801 (20.2%).

**Table 1:** Characteristics of subjects responding to EQ-5D-Y descriptive system (*N* = 8919)

Variables	n (%)
Age (years), Mean (SD)	10.2 (1.8)
Sex (male) (out of *n* = 8557, *n* = 362 missing)	4397 (51.4)
School grade (out of *n* = 8918, *n* = 1 missing)	
1	2302 (25.8)
2	1324 (14.9)
3	1373 (15.4)
4	1316 (14.8)
5	1225 (13.7)
6	1378 (15.5)
Diagnosis of asthma, ever	533 (6.0)
Wheezing or whistling in the chest in the past 12 months	256 (2.9)
Treatment of asthma in the past 12 months	163 (1.8)
School absence due to asthma in the past 12 months	41 (0.5)
ER visit due to asthma in the past 12 months	34 (0.4)
Diagnosis of allergic rhinitis, ever	3272 (36.7)
Sneezing or a runny or blocked nose in the past 12 months	3695 (41.4)
Treatment of allergic rhinitis in the past 12 months	2477 (27.8)
School absence due to allergic rhinitis in the past 12 months	47 (0.5)
Diagnosis of eczema (atopic dermatitis), ever	2002 (22.5)
Itchy rash in the past 12 months	1801 (20.2)
Treatment of eczema in the past 12 months	950 (10.7)
School absence due to eczema in the past 12 months	38 (0.5)

The missing proportion of the EQ-5D-Y for all respondents and by actual responders of the EQ-5D-Y are shown in the Table 2. Of the 9117 responses, 198 (2.2%) had missing data for at least one dimension of the EQ-5D-Y, and the missing proportion for each dimension ranged from 155(1.7%) in ‘looking after myself’ dimension to 162 (1.8%) in the ‘feeling worried, sad, or unhappy’ dimension. There were 1258 (13.8%) missing responses on the EQ-VAS. There was no invalid answer on the EQ-5D-Y descriptive system or EQ-VAS. Of the respondents of EQ-5D-Y, 3627 (39.8%) were the students with the help of their parents, followed by 3371 (37.0%) parents and 1443 (15.8%), the students themselves. When students themselves responded, the missing proportion was somewhat lower, but not significantly.

**Table 2:** Non-response proportion in EQ-5D-Y descriptive system and EQ-VAS according to actual respondents

		Actual respondents of the EQ-5D-Y			
	All (*n* = 9117)	Parents (guardians) (*n* = 3371)	Students with parents’ assistance (*n* = 3627)	Students themselves (*n* = 1443)	Who did not respond to the actual respondents (*n* = 676)
EQ-5D-Y dimensions					
Mobility (walking about)	1.73	0.47	0.36	0.14	18.79
Looking after myself	1.70	0.47	0.25	0.14	18.93
Doing usual activities	1.72	0.47	0.25	0.14	19.23
Having pain or discomfort	1.71	0.44	0.28	0.07	19.23
Feeling worried, sad or unhappy	1.78	0.50	0.33	0.28	19.08
EQ-VAS	13.80	13.02	5.18	4.37	84.02

Responses with missing values for the EQ-5D-Y descriptive system (198 cases, 2.2%) were excluded from the analysis. The remaining 8919 cases (97.8%) were used for the final analysis.

The proportions of reported problems by dimension and EQ-VAS score by grade (low vs. high grade) are summarized in Table 3. The proportion of reported problems in the 4th dimension ‘having pain or discomfort’ and in the 5th dimension ‘feeling worried, sad, or unhappy’ was higher than in the remaining first three dimensions. The mean VAS score was 91.2 (SD, 11.1) in grade 1st through 3rd, and 88.2 (SD, 13.0) in grade 4th through 6th. A total of 71 distinct EQ-5D-Y health states were reported. Among EQ-5D-Y health states, ‘11111’ (i.e., full health) states accounted for 82.0%, followed by the ‘11121’ and ‘11112’ health states at 5.0 and 4.8%, respectively (data not shown).

**Table 3:** Reported problem and VAS score in the EQ-5D-Y by dimension and grade group

EQ-5D-Y dimensions	Grade (1-3) *N* = 4999	Grade (4-6) *N* = 3919	*P*-value^*^
	n (%)	n (%)	
Mobility (walking about)			
No problems	4916 (98.3)	3817 (97.4)	0.005
Some problems	78 (1.6)	92 (2.4)	
A lot of problems	5 (0.1)	10 (0.3)	
Looking after myself			
No problems	4828 (96.6)	3874 (98.9)	< 0.001
Some problems	166 (3.3)	41 (1.1)	
A lot of problems	5 (0.1)	4 (0.1)	
Doing usual activities			
No problems	4828 (96.6)	3806 (97.1)	0.334
Some problems	160 (3.2)	107 (2.7)	
A lot of problems	11 (0.2)	6 (0.2)	
Having pain or discomfort			
No problems	4596 (91.9)	3491 (89.1)	< 0.001
Some problems	395 (7.9)	418 (10.7)	
A lot of problems	8 (0.2)	10 (0.3)	
Feeling worried, sad or unhappy			
No problems	4590 (91.8)	3533 (90.2)	0.016
Some problems	393 (7.9)	375 (9.6)	
A lot of problems	16 (0.3)	11 (0.3)	
VAS, n	4391	3491	
VAS, mean (SD)	91.2 (11.1)	88.2 (13.0)	< 0.001

^*^P-values by Fisher’s exact test or independent t test

Comparisons of reported problems and VAS scores on the EQ-5D-Y by allergic symptoms are shown in Table 4. There were significant differences in the problem reporting proportion in all EQ-5D-Y dimensions between groups with and without allergic symptoms. There was a particularly large difference in problem reporting rates in ‘having pain or discomfort’ and ‘feeling worried, sad or unhappy’ dimensions compared with the other dimensions of the EQ-5D-Y. Among the allergic symptoms, the group experiencing symptoms of wheezing or whistling in the chest reported significantly more problems than the group without it. The problem reporting rate in the group experiencing wheezing or whistling in the chest in the respective dimensions was 21.1% in ‘having pain or discomfort’ and 18.8% in ‘feeling worried, sad or unhappy’. The average of EQ-VAS score for children experiencing wheezing was 84.5, for runny or blocked nose 88.8, and for itchy rash 88.6, respectively. Students with allergic symptoms had a lower EQ-VAS scores compared to those without. There were similar patterns of problem reporting of the EQ-5D-Y by the diagnosis and treatment of allergic diseases (data not shown).

**Table 4:** Reported problems and VAS score on EQ-5D-Y by allergic symptoms

EQ-5D-Y dimensions	Allergic symptoms in the past 12 months								
	Wheezing or whistling in the chest			Runny or blocked nose			Itchy rash		
	Yes (*n* = 256)	No (*n* = 8663)	*p*-value^*^	Yes (*n* = 3695)	No (*n* = 5224)	*p*-value^*^	Yes (*n* = 1801)	No (*n* = 7118)	*p*-value^*^
	n (%)	n (%)		n (%)	n (%)		n (%)	n (%)	
Mobility (walking about)									
No problems	246 (96.1)	8488 (98.0)	0.037	3603 (97.5)	5131 (98.2)	0.021	1745 (96.9)	6989 (98.2)	<.001
Any problems	10 (3.9)	175 (2.0)		92 (2.5)	93 (1.8)		56 (3.1)	129 (1.8)	
Looking after myself									
No problems	242 (94.5)	8461 (97.7)	0.001	3591 (97.2)	5112 (97.9)	0.042	1742 (96.7)	6961 (97.8)	0.008
Any problems	14 (5.5)	202 (2.3)		104 (2.8)	112 (2.1)		59 (3.3)	157 (2.2)	
Doing usual activities									
No problems	241 (94.1)	8394 (96.9)	0.013	3538 (95.8)	5097 (97.6)	<.001	1718 (95.4)	6917 (97.2)	<.001
Any problems	15 (5.9)	269 (3.1)		157 (4.3)	127 (2.4)		83 (4.6)	201 (2.8)	
Having pain or discomfort									
No problems	202 (78.9)	7886 (91.0)	<.001	3248 (87.9)	4840 (92.7)	<.001	1530 (85.0)	6558 (92.1)	<.001
Any problems	54 (21.1)	777 (9.0)		447 (12.1)	384 (7.4)		271 (15.1)	560 (7.9)	
Feeling worried, sad or unhappy									
No problems	208 (81.3)	7916 (91.4)	<.001	3266 (88.4)	4858 (93.0)	<.001	1582 (87.8)	6542 (91.9)	<.001
Any problems	48 (18.8)	747 (8.6)		429 (11.6)	366 (2.1)		219 (12.2)	576 (8.1)	
VAS, n	216	7597		3274	4539		1608	6205	
VAS, mean (SD)	84.3 (17.2)	90.1 (11.0)	<.001	88.8 (13.1)	90.7 (11.2)	<.001	88.6 (13.1)	90.2 (11.8)	<.001

^*^P-values by Chi-square test or independent t test

Finally, the proportions of reported problems and VAS scores on the EQ-5D-Y by the number of allergic symptoms are shown in Table 5. As the number of allergic symptoms increased, the problem reporting rate tended to increase in all dimensions. In particular, the problem reporting rates in having pain or discomfort’ and ‘feeling worried, sad or unhappy’ dimensions increased significantly. As the number of allergic symptoms increased, the EQ-VAS score decreased significantly.

**Table 5:** Reported problems and VAS score on EQ-5D-Y by the number of allergic symptoms reported

EQ-5D-Y dimensions	The number of allergic symptoms reported in the past 12 months					
	0 (*n* = 4463)	1 (*n* = 3253)	2 (*n* = 1110)	3(*n* = 93)	*p*-value^*^	Post hoc grouping^a^
	n (%)	n (%)	n (%)	n (%)		
Mobility (walking about)						
No problems	4388 (98.3)	3186 (97.9)	1072 (96.6)	88 (94.6)	< 0.001	0 > 2
Any problems	75 (1.7)	67 (2.1)	38 (3.4)	5 (5.4)		
Looking after myself						
No problems	4372 (98.0)	3174 (97.6)	1070 (96.4)	87 (93.6)	0.001	0 > 2
Any problems	91 (2.0)	79 (2.4)	40 (3.6)	6 (6.5)		
Doing usual activities						
No problems	4362 (97.7)	3135 (96.4)	1052 (94.8)	86 (92.5)	<.001	0 > 1,2,3
Any problems	101 (2.3)	118 (3.6)	58 (5.2)	7 (7.5)		
Having pain or discomfort						
No problems	4174 (93.5)	2914 (89.6)	934 (84.1)	66 (71.0)	<.001	0 > 1 > 2 > 3
Any problems	289 (6.5)	339 (10.4)	176 (15.9)	27 (29.0)		
Feeling worried, sad or unhappy						
No problems	4168 (93.4)	2932 (90.1)	948 (85.4)	76 (81.7)	<.001	0 > 1 > 2,3
Any problems	295 (6.6)	321 (9.9)	162 (14.6)	17 (18.3)		
VAS, n	3860	2888	985	80		
VAS, mean (SD)	90.9 (11.0)	89.4 (12.6)	87.9 (13.5)	84.3 (18.1)	<.001	0 > 1 > 2 > 3

^*^P-values by Chi-square test or ANOVA test

^a^Multiple comparisons with Bonferroni correction (significance level = 0.05/6 = 0.0083)

## Discussion

This study compared HRQOL according to allergy symptoms in Korean elementary school students and focused on the importance of school-based allergic disease management. Our study is a large population-based sample study. This study showed that the use of EQ-5D-Y to measure the quality of life of allergic children in elementary school students was feasible and showed construct validity with allergic symptoms.

We compared the quality of life measured with EQ-5D-Y in students with allergic symptoms versus those without. As expected, the quality of life measured with EQ-5D-Y was significantly lower in students with allergic symptoms than in those without, and the quality of life tended to decrease as the number of allergic symptoms increased. Raven-Sieberer et al. [20] indicated that the EQ-5D-Y might not be very useful for discriminating between respondents in the general population. However, our results indicate that EQ-5D-Y can be useful in distinguishing the quality of life of respondents in the general population.

In the EQ-5D-Y descriptive system, we found a high prevalence of problems reported of ‘having pain or discomfort’ and ‘feeling worried, sad or unhappy’ dimensions in all allergic symptoms. Students with wheezing symptoms reported the most problems in all dimensions of EQ-5D-Y, and also the lowest VAS scores. Thus, among the investigated allergic diseases, asthma appears to have a major effect on the HRQOL of children. Trends in the EQ-5D-Y problem reporting rate and EQ-VAS score in this study were similar depending on whether the allergic diseases were diagnosed or not, although not shown in the results. Covaciu’s study reported that allergic diseases, and particularly asthma, significantly affect children’s HRQOL. Frequent wheezing is associated with a high prevalence of problems of ‘pain or discomfort’ and ‘anxiety or depression’ dimensions (26.7 and 23.3%, respectively) [5]. This is similar to our findings, although there is a difference in the quality of life of children assessed by parents using the EQ-5D-3 L. Coviciu et al. reported that allergic comorbidity was associated with the EQ-VAS score [5]. In a Swedish study evaluating the quality of life of patients with asthma with EQ-5D-Y, the problem reporting rates of dimensions of ‘doing usual activities’ was 20%, ‘having pain or discomfort’ was 33.3%, and ‘feeling worried, sad or unhappy’ was 20%, and the VAS average score was 80.7 [6]. Compared with our study, the problem reporting rate is higher in the ‘doing usual activities’ dimension (20% versus 5.9%) [6]. The subjects of the study in Sweden were recruited from the clinics of Stockholm, so the asthma symptom of the subject is likely to be worse than the school-based survey [6]. Our findings are consistent with studies that reported adversely affected quality of life in patients with allergic rhinitis [4] or atopic dermatitis [3, 30].

Of the total respondents, 2.2% did not answer at least one EQ-5D-Y question, and 13.8% did not respond to the EQ-VAS. A multi-national study [20] reported that the proportion of missing responses in the EQ-5D-Y dimensions ranged from 0% in Spain and Italy to 2% South Africa, and non-responders of the EQ-VAS ranged from 0% in Italy to 9% in Germany. Our study is a survey done at the student’s home with written instruction, while the multi-national study was mainly conducted at the school, and in Italy, the researchers provided assistance if needed [20]. Our study was not a face-to-face survey, so we recorded the person who wrote the EQ-5D-Y. More than half were written by students with or without assistance. However, 37% of respondents are parents, and the lower the grade level, the higher the percentage of parents answering was. Jelsma also reported more missing responses in the lower grades [19]. Parents’ and children’s thoughts may be different in evaluating the quality of life of a child [11–13]. Therefore, the researcher would be wise to assist the elementary school students, especially in the lower grades, to reduce the missing values and improve the completeness of the data.

The strength of our research is that we have covered a large general population. However, there are several limitations of this study. First, our study is a cross-sectional questionnaire survey without clinical examination, so there may be a limitation on the accuracy of diagnosis. However, in previous allergic diseases prevalence studies in elementary school students in Korea, prevalence rates over the past 12 months of symptoms of asthma ranged from 4.8 to 6.5%, allergic rhinitis from 32.9 to 38.5%, and atopic dermatitis from 14.5 to 16.7% [27, 31]. These findings were not much different from our study. Second, our findings include 37% of parents’ responses to EQ-5D-Y. We conducted the same analysis with students only if they wrote their own and with help. The results were not significantly different from those for the whole. In some cases, the *p*-value slightly increased, but still remained significant. Third, our study could not evaluate the convergent validity, responsiveness and reliability of the EQ-5D-Y due to limited questionnaires and the study design without retest. Further studies are required to examine the other psychometric properties such as test–retest reliability, and responsiveness of the Korean EQ-5D-Y for elementary school students with a wider range of clinical conditions.

## Conclusions

In conclusion, the HRQOL was lower in the students with allergic symptoms compared those without, and the HRQOL became lower as the number of symptoms increased. The EQ-5D-Y instrument is acceptable in terms of feasibility and construct validity in assessing the quality of life of elementary school students in Korea. However, it would be beneficial for the researcher to assist the elementary school students, especially in the lower grade, when they fill out the questionnaire to reduce missing values and improve the completeness of the data. EQ-5D-Y would be a useful tool for measuring the quality of life of students with allergic symptoms.

## Abbreviations

EQ-5D-3L: EQ-5D 3 level version
EQ-5D-Y: EQ-5D for youth
HRQOL: Health-related quality of life
ISAAC: International Study of Asthma and Allergies in Childhood
